# Western Texas Medical Association—Regular Meeting August, 31, 1899*Reported for Texas Medical Journal, official organ.

**Published:** 1899-10

**Authors:** 


### Society Notes.

### Western Texas Medical Association—Regular Meeting August, 31, 1899.*

*Present:* Drs. Jno. S. Lankford, Robt. E. Moss, B. F. Kingsley, B. E. Hadra, A. S. McDaniel, S. Burg, E. Clavin, J. H. Bindley, J. D. Forest, and Wm. E. Luter, of San Antonio; Dr. H. A. Tutweiler, of Flatonia; Dr. Jno. S. Turner, of the Southwestern Insane Asylum; Dr. G. H. Morre, of San Antonio, Mr. Albert E. Hyde, of Chattanooga, Tenn., and Mr. Ed. Braden of this city.

President Dr. Jno. S. Lankford presided; Dr. Wm. E. Luter, secretary.

The minutes of the previous meeting were read, and, upon motion, approved. The committee appointed to consider the advisability of uniting the Austin District Medical Society with the Western Texas Medical Association was continued, as was also the Committee on Streets.

The Board of Censors reported favorably on the application of Dr. J. P. Magnus, of Devine, and, upon motion of Dr. S. Burg, Dr. Magnus was unanimously elected to membership of the Association.

The application of Dr. G. H. Morre was presented, and referred to the Board of Censors.

The question of our local Association being auxiliary to and having a representation in the State Association, necessitating the payment of annual dues to the State Association, was brought up, and, upon motion, President Dr. Jno. S. Lankford was requested to obtain information in regard to the matter, and act as he thought best.

In speaking of the health of our city, it was asked what had become of our board of health, and a motion was made by Dr. Hadra

*Reported for Texas Medical Journal, official organ.

to the effect that we request the mayor and the city council to appoint a board of health. The motion was carried.

Dr. Kingsley introduced the following resolution, which was unanimously endorsed by the Association:

WHEREAS, It has come to the knowledge of the Western Texas Medical Association that there has been enacted by the Twenty-fifth Legislature of Texas, a law for the regulation of plumbing and drainage, which aims to take out of the domination of municipal politics the care of the people's health in this important department; and,

WHEREAS, It is the policy of all reputable medical men to encourage all methods looking to the betterment of public health along the lines of preventive medicine; therefore, be it

*Resolved,* That it is the sense of the Western Texas Medical Association that the Act of September, 1897, regulating the plumbing industry, and creating examining boards, operated under the direct supervision of health boards, be endorsed, and that we earnestly urge the cities of Texas to ratify at once this wise sanitary measure, believing that its purpose is for the betterment of the public health.

Dr. Kingsley said that Mr. Albert E. Hyde, of Chattanooga, Tenn., an eminent sanitarian, was in the city, and could be conveniently spoken to, and he suggested that Mr. Hyde be invited to address our Association on "Sanitation," which suggestion was carried out. While awaiting Mr. Hyde's arrival, Dr. B. E. Hadra reported an exceedingly interesting case of

### VOLVULUS OF SMALL INTESTINE AND A SUCCESSFUL RESECTION OF THE GUT.

Dr. Hadra said:

Having only recently been informed of my duty to read a paper before you, I must ask you to accept merely a report of a surgical case with some additional remarks, which I hope may prove sufficiently interesting for discussion. Mrs. T., Irish, a farmer's wife, 26 years old, mother of several children, an invalid for the past four or five years. She has never been seriously sick; had more or less easy deliveries. A trouble, indistinct as to its situation, and growing worse steadily, rendered her an invalid, unable to attend to her duties. She designates her whole left abdominal side as the seat of more or less pains, worse on bending and stooping, sometimes very sharp, but she cannot locate them at any certain point. Her appetite is very poor, and a nauseating feeling very frequent. Her bowels though, always were more or less regular; has had no fever recently.

As a matter of course, she has been under treatment, her womb serving mainly as the target of surgical aggressiveness. Examination by vagina met a firmly adherent retroflexion with multiple cervical lacerations; parametria hard to reach by palpation on account of the retroflexion, but seem to be free of abnormal contents; parts not very tender. Abdomen examined in a methodical way offers no indication of a disease pertaining to the stomach, gall-bladder, liver, kidneys, appendix, etc., only when pressing on the left rectus muscle opposite the umbilicus a great tenderness is encountered, the muscle becomes contracted by reflex, the palpating finger feels an indistinct resistance and percussion renders questionable abdominal dullness. Below the level of this point and over the pelvic region nothing abnormal is found. Patient is directed to take a good purgative. When examined again, the same conditions were met; the resistance more pronounced, and more distinct swelling felt. She now locates her suffering unhesitatingly at this point, and insists that all of her trouble starts there, extending mostly towards the stomach. A third examination was made a few days later with the same result. The trouble was then considered located in the bowel, though the retroflexion and parametritis were not left out of the calculation as co-incidental factors of suffering.

The diagnosis as to the nature of the supposed bowel trouble seemed to be doubtful. A carcinoma is at once excluded by the long duration, but I have seen a form of fibro-sarcoma lasting that long, and longer. It could be also a pure fibroma or a lipoma, which has been observed in the bowel walls by several writers. Whatever new growth it was, it must have been situated outside of the lumen, or only partially occluded it, as the bowels act regularly. Thus the seat of it was most likely in the mesentery. But it also could be a chronic abscess walled off by adhesions, and even a foreign body may have been thought of. A looping or intussusception was excluded on account of the unimpaired passage of fæces.

Thus the assumption of a tumor-like growth in connection with the surface of the bowel or with the mesentery seemed to be most probable. Operation was advised, and the proposition accepted. It was performed on the 7th of July, with the kind assistance of Drs. Burg, Edmonson and Paschal. A longitudinal incision of about two inches in length was made over the involved location through the middle line of the left rectus muscle, about one inch to the left of the umbilicus. The small intestine presenting was examined with the finger, and not offering much enlightenment it was pulled out through the incision. Now, one could see that it was highly injected, and when a sufficient amount of it was brought to light it plainly became visible that a portion of the small intestine was about double the calibre of the portions below and above, and that the walls were very much thickened. Then at either end of this so changed gut a very distinct demarcation line, or rather a circular impression mark, was observable. While the intestinal loop was so exposed in a straightened out position, the color of the swollen portion became more and more natural. Now, there was no question, but that we had before us a piece of small intestine that had been looped on itself, and thus partially constricted, and that this condition had caused its venous stasis, and the hypertrophy of its walls. As a careful insertion of the finger did not further enlighten me as to the mechanical conditions that may have led to the volvulus, and as the abdominal cavity could be kept perfectly closed during the operation, we thought best not to go any further in our investigation. In the involved locality, in the middle of the left side of the abdomen an internal hernia is not to be expected; neither would the urachus be found there, and in the absence of adhesive bands it would have been nothing but unjustified gratification of curiosity if I had widened the incision and examined the abdominal cavity.

But what must be done now? A simple reposition after straightening out the bowel seemed not sufficient to guard against a repetition of the twisting. Attaching the gut to the parietal peritoneum might become the source of new trouble. Thus, after all, it seemed best, or rather the most reasonable thing under the given circumstances, having to deal with a woman who had to work, and who could not spend years under medical observation, and spend months for another surgical experiment, to do a radical operation, and resect the piece of gut between the two compression marks. It was done, and a Murphy button used for an end-to-end anastomosis. The button was perhaps too small, and a good deal of puckering made the job look a little awkward.

The mesentery was tied off in four or five small portions in a horizozntal line, cut off, and then the cut line halved and stitched together like two parellel lines of cut surface. The omentum was carefully spread out over the surface of the bowel. The incision was then closed by two rows of catgut, one for the peritoneum and one for the outer fascia, and three general wire sutures.

Patient suffered from chloroform nausea more than ordinarily for several days. Temperature rose to 102, and on the fourth day tenderness and general malaise made close inspection of the abdomen necessary. There was no distension, but the region of the incision was swollen and reddened, and a very plain cutaneous emphysema in the left groin, later extending into the thoracic region, made things look very ugly. There was at once given an escape to the pus which had formed in the abdominal wall, and as the pus did not smell of fæcal matter, I felt somewhat relieved. I do not know, even today, to what to attribute that emphysema; whether to a leak in the gut, or to the mean catgut, which no doubt was not aseptic. There was a good movement from the bowels on the fourth day, flatus were regularly passing, and there was no symptom of peritonitis; that much against the leakage theory. On the other hand the putrefactive gas-producing bacteria are rarely met in catgut to my knowledge, and if they are, I think they generally are more dangerous in their effects than they were in our case. Whatever it was, she got along nicely; the pus was draining well, stool became regular, and though the emphysema persisted, she was apparently well towards the twelfth day. The button had come away by that time also. After a week's respite she got feverish again, her temperature running up to 103, but a few doses of antifebrine mended that promptly. She then slowly got stronger, begun to eat ravenously; in short, she got perfectly well and free of all her trouble.

In reviewing this case many points seem to be of interest, and none more than the question, how the twisting of the bowel, which I think unquestionably the trouble, occurred. The only solution I can offer, is that an existing abnormal disproportion of the length of the bowel and the width of its mesentery, or, in other words, a too long bowel, had led to the looping, some time, years ago, when occasionally extraordinary active peristalsis became excited by some unknown cause.

It is very difficult to find in literature and in text-books any satisfactory statement as to this disproportion, though the named discrepancy between the bowel and mesentery is generally admitted as a possible cause of twisting. In our case I must say I had no idea of the length of the bowel removed until I measured it after the operation. It amounted to four and one-half feet, while the line of ablation of the mesentery very close to the bowel, seemed to me not more than about three-fourths of a foot. The gut was very much folded, and when straightened out appeared only of half the previous calibre. The obstruction was evidently not complete, as the regularity in defæcation proved, but every time the chymus had to overcome the twist, an excessive peristaltic action of the bowel was required, which must have caused quite vehement pains.

Another point which interests me very much, and which I have at different times called your attention to, is the inability of the patient to locate abdominal pains, a fact we see so frequently in appendicitis. Also here, not until the mind had been directed to the true position of the trouble, did the patient give a definite account of the locality, while afterwards, the most positive and unerring statement was made. Let me also once more take occasion to mention the so much misconstrued and misunderstood form of so-called neurosis of the stomach, which goes as a disease *sui-generis* with the stomach specialist, the want of appetite coupled with a real abhorrence of taking food. The disease is called, as you know, "orexia." I do not hesitate to express my opinion that such cases are only very seldom symptoms of a true nervous disease of the stomach, but mostly such are palpable disorders of that organ, and still oftener of the bowels. They are reflex troubles, and a search for the real cause should never be omitted. The whole case is an excellent illustration of the necessity of a systematic examination of the whole abdomen and pelvis in every ailment which is not plain to the eye or touch, and especially in female diseases this rule holds good. How many so-called gynecological cases have been unmasked since our better knowledge. Preëminently, appendicitis has taught us that not everything a woman has to complain of is her "womanhood."

In the discussion which followed on Dr. Hadra's report, Dr. Kingsley said that this was one of the few successful cases of intestinal anastomosis, witnessed at San Antonio, and it shows the result of prompt scientific treatment and procedure. In the use of the Murphy button Dr. Hadra thought it was best to use the smallest button consistent with the calibre of the gut, even though there was considerable puckering.

Dr. Luter reported a case coming under his care where there was a suddenly developed tumefaction in the left lumbar region which could be clearly and definitely mapped out. General abdominal pain, more pronounced over the tumor, nausea and vomiting, marked depression, with rather *free* evacuations, were some of the most important symptoms.

The hardness in the left lumbar region lasted for some three or four days, when it disappeared as suddenly as it came. No purgatives were allowed, and the case was treated with high enemas.

At the request of the Association, Mr. Albert E. Hyde now gave a valuable talk on

"SANITATION AND SANITARY PLUMBING,"

and set forth the necessary precautions to be taken in keeping the city clean. He dwelt at length on plumbing to be found in connecting with the sewers, and spoke of the necessity of having a competent inspector of plumbing. Texas, he said, was the first State south of the Ohio river, to pass an act removing the regulation of sanitation from politics. His address was full of valuable suggestions, and upon its conclusion the Association tendered him a vote of thanks.

Dr. Hadra made a valuable talk, in which he plainly set forth

HOW DISEASES CAN BE CONTRACTED THROUGH TELEPHONES, and suggested that some remedy be taken to prevent this, as a person using a public telephone is very liable to inhale the germs of tuberculosis. A number of preventives were suggested, and examples cited how the dangers might be overcome.

Dr. Kingsley thought public telephones were dangerous, and told of an invention of Dr. L. L. Lacy, of Austin, formerly of San Antonio, whereby, with an electric light in the mouthpiece of the telephone, he claims to destroy the disease germs.

Dr. Kingsley reported the three following interesting cases. Not only on account of their intrinsic interest, but on account of their occurrence within the period of a month in my practice, I desire to report the three following interesting cases. I can do so only briefly, on account of other papers, and the fact that each of the subjects might consume the attention and discussion of the Association for an entire evening:

NO. I.—COMPLETE, OR PLACENTA-PRAEVIA CENTRALIS.

This complication of pregnancy is indeed so rare that in twenty-five years of practice I have only seen two cases, and many physicians have *never* met with it. For the purposes of this report I shall recognize only two forms, *P. Centralis,* and *P. Lateralis; P. Centralis* where the placenta is implanted directly over the internal os, and *P. Lateralis* where the implantation is on the sides, a margin only being over the os internum. Of the latter I have seen quite a number. Pinord, in 15,000 confinements, never saw a case of central placenta-prævia. According to various authors the average frequency of both forms is 1 to 1000. So much for their rarity. Other recognized pathological conditions which complicate these conditions, especially in the third stage, is the tendency of the placenta to become adherent to the uterine walls. The tough and thickened membranes. The principal symptom is hemorrhage coming on at any time in the latter part of pregnancy. The earlier it comes on, the os and cervix are nearer normal in appearance and to the touch. The nearer full-term, the larger and softer the os; coming on at term, or shortly before, the parts are usually dilated or dilatable. It is a condition that needs prompt action on the part of the accoucheur in recognizing the difficulty, and in dealing with it.

On July 23rd, was called to see Mrs. B., age 22 years, about to be confined with her second child; first confinement in every way normal. I learned that she had passed quite a large clot, and that from time to time for two or three months she had passed some blood. Upon examination, I found the os well dilated, and a soft mass presenting above, and no part of the child to be felt; nor could I determine from whence came the hemorrhage. My suspicions were aroused. I packed the vagina with iodoform gauze, and directed that I be sent for at once should hemorrhage recur. On the 24th, the next noon, I was called to the telephone and asked what should be done for her fainting spells. I inquired if she had been bleeding; the reply was, that she had, more or less of the time since the evening before. I asked Dr. Jackson to accompany and assist me in the case. His examination confirmed my diagnosis of *placenta praevia centralis,* and he heartily endorsed my determination to empty the womb as soon as possible. The patient had not felt fœtal life for two days. The instruments and hands were carefully prepared, the patient chloroformed, the finger swept around the internal os, and between the placenta and uterine wall, I found no detachment anywhere; so the placenta was perforated with the finger, and rapid dilatation made through which the vertex of the child could be felt. As soon as possible I applied forceps and brought the head into the lower uterine segment, and by pressure against the uterine and pelvic walls controlled the hemorrhage. Finding it impossible to deliver in this position, I removed the forceps, introduced my hand and succeeded in turning and delivering by the feet in a few minutes. The placenta was removed by piece meal, and was adherent only in one place to the left. To be certain that no placental tissue or membrane remained I used a large dull curette, washed the womb out with a bichloride solution, 1 to 3000, and packed uterus and vagina with five per cent. iodoform gauze, which I removed in fourteen hours. No laceration occurred, and the patient made an uninterrupted recovery.

NO. II.—ANTE-PARTUM CONVULSIONS.

Mrs. C., aged 20, married one year, pregnant six months, was seen by me in consultation with Dr. Hicks, who had charge of the case, August 7th, 12 m. Several physicians had seen the patient during the forenoon, and advised palliative treatment. She had been perfectly well until within two weeks, during which time she had had some headache, and slightly swollen limbs. Her pulse was 96 to 102, rather full and not very compressible, the urine was heavily charged with albumen, and scant. In this connection, I may say that I have never seen a case of *ante-partum* or *post-partum* convulsions in which there was not albumen present, though they do occur, and usually the pulse is full and non-compressible, showing great arterial tension.

Between early morning and 10 a. m. she had four convulsions, during which time she had been given calomel, enemas, chloroform, pilocarpine and salines, which were now acting vigorously. Between 12 and 1 p. m. she had two more convulsions, and it was decided to empty the womb at once. Since the first one she lay in a semi-conscious state. She was given veratrum viridi hyperdermically m 10, repeated twice, an hour apart with six and five m, and the pulse brought down to about 50. The cervix was rapidly dilated with fingers and dilator, the short forceps applied, and the child delivered. The after-birth was easily detached, and seemed to be entire, but on account of possible unavoidable traumatism it was deemed wise to pass a dull curette over the cavity, wash out with 1 to 3000 bichloride solution, and pack lightly with iodoform gauze. Time under chloroform, two hours; entire time, three hours. No more convulsions occurred; an antiseptic pad was used, a milk diet and Stafford water, and barring a little fever on the third and fourth day, she made a prompt and easy recovery.

I attach great importance to the use of veratrum in these cases. I believe it is the sheet anchor. I do not mean to say this remedy alone will save these patients, but that it is of primary importance. Its *modus operandi* is that of a vaso-dilator, and thus bleeding the patient into her own veins and relieving cerebral congestion. Of mechanical importance, emptying and freeing the womb of all its contents under the most rigid antiseptic precautions at all times stands first. This having been done, the exhibition of veratrum stands next. The administration of chloroform, for whatever purpose, does not contraindicate the use of veratrum. The indication for its use and its repetition and continuance, is the pulse; with it, the pulse can in short order, be brought down to 40 and 50 and kept there. I am almost ready to assert, the womb being empty, it is impossible for a convulsion to occur, when its effect is well established. I have never lost a case in sixteen years since I began its use, and with an experience of some twelve cases, and in the practice of others who depend largely on it, some eight or ten more. I have thus seen or known of about twenty cases treated with this remedy without a death.

NO. III.—ECTOPIC PREGNANCY.—OPERATION.

I was summoned, on August 9th, to a distant town to see a lady whom I was informed was very sick. I arrived about noon, August 10th, and found a young married woman who had borne two children; the last, sixteen months ago, which she was still nursing. Menses had been regular since eight months after confinement, until last one, which had gone over twelve days. In order to bring it on she had used an injection of hot water a day or two previously. On the morning of the 9th of August she was suddenly seized with an acute cutting pain in her left side; she grew pale, became nauseated and vomited; her pulse was very feeble and irregular. These symptoms had continued, were present when I saw her thirty-six hours afterward. There was also a distressed countenance, shallow and quick respiration, constant vomiting and restlessness, and a fluttering and feeble pulse. My diagnosis was an ectopic pregnancy with steady hemorrhage. Towards evening it became apparent to me that if anything was to be done to try and save her, it must be done soon. After consultation, an operation was agreed to and proceeded with at once. Upon opening the abdomen, blood poured out in great quantities. I introduced my hand and turned out clot after clot from different parts of the abdomen, and quickly clamped the broad ligament and brought it to view, when a perforation in the tube was at once apparent as seen in the specimen presented; the tube was clamped on either side of the perforation, and it was tied off with strong catgut; the abdomen thoroughly washed out with normal salt solution, a quantity being left in, and the abdomen closed, consuming about thirty-five minutes; the pulse was good after the operation. No transfusion apparatus, which might still have saved her life, being at hand, copious rectal injection of hot normal solution was given, but she became gradually pulseless, and died about sixteen hours after the operation.

When called to a case of ectopic pregnancy, we may be confronted with a sudden onset of symptoms, without any previous warning, as in this case, where the symptoms plainly denoted internal hemorrhage, or we may more likely have a history of one or two periods missed, with symptoms like those described, but of a much milder character, and passing off and repeated at intervals of a few weeks. Wherever these symptoms are long continued an operation is urgently indicated. We undoubtedly have abdominal, ovarian, tubal and interstitial forms of ectopic pregnancy, and upon the age of the fœtus and its location, as well as upon prompt recognition and early operation, will depend the life of the patient, and if diagnosed early these cases are not fraught with the same danger or mortality as when the fœtus develops to the third or fourth month. Up to, and including the second month, most cases can be successfully dealt with through the abdominal operation; after this period, one has to be governed by conditions and circumstances in dealing with them. The greater one's experience in ectopic pregnancy, the greater his confidence in his ability to make a correct diagnosis at all times.

Dr. A. S. McDaniel reported having one case of *placenta-praevia centralis,* in which he promptly emptied the uterus, but the patient died in forty-eight hours after the operation.

Dr. Hadra, in his many years of practice, has met with only three cases of *placenta-praevia centralis,* and was successful in saving one of them.

Drs. Robert E. Moss and Sigmund Burg were appointed essayists for the next meeting, which will be Thursday, September 28th.

